# Limited contributions of bacteria and fungi to coral nutrition revealed by amino acid δ^13^C analysis

**DOI:** 10.1038/s42003-025-08888-x

**Published:** 2025-10-27

**Authors:** Qifang Wang, Jiachen Li, Xijie Zhou, Lingfeng Huang, Tuo Shi, Tiantian Tang, Jonathan Y. S. Leung, Xinqing Zheng

**Affiliations:** 1 Key Laboratory of Marine Ecology Conservation and Restoration, Third Institute of Oceanography, Ministry of Natural Resources, Xiamen, China; 2 Key Laboratory of the Ministry of Education for Coastal and Wetland Ecosystems, College of the Environment and Ecology, Xiamen University, Xiamen, China; 3 Observation and Research Station of Island and Coastal Ecosystem in the Western Taiwan Strait, Ministry of Natural Resources, Zhangzhou, China; 4 Observation and Research Station of Wetland Ecosystems in the Beibu Gulf, Ministry of Natural Resources, Xiamen, China; 5 State Key Laboratory of Marine Environmental Science, Xiamen University, Xiamen, China; 6 Marine Genomics and Biotechnology Program, Institute of Marine Science and Technology, Shandong University, Qingdao, China; 7 Guangdong Provincial Key Laboratory of Marine Disaster Prediction and Prevention, Shantou University, Shantou, China; 8 Ningbo Institute of Oceanography, Ningbo, China

**Keywords:** Stable isotope analysis, Animal physiology, Tropical ecology

## Abstract

Corals often form reef ecosystems that support diverse marine life, but they are sensitive to environmental fluctuations that can affect their nutrient acquisition. While coral-associated microbes (e.g., Symbiodiniaceae, bacteria and fungi) may supplement nutrients to coral hosts via metabolite translocation and nutrient recycling, the extent to which these microbial partners contribute to coral autotrophy or heterotrophy remains unclear. Here, we seasonally measure the carbon isotopes of amino acids (δ^13^C_AA_) in reef-building coral *Pocillopora damicornis* and its nutrient sources (e.g., Symbiodiniaceae and particulate organic matter). Regional Bayesian mixing models show that *P. damicornis* increased autotrophy (from 67.1 to 80.5%), but decreased particulate feeding (from 32.9 to 19.5%) from the cool season to the warm season. Stable essential δ^13^C_AA_ values (valine, leucine and isoleucine) suggest limited seasonal changes in microbial contributions. Linear discriminant analysis, which combines current and published data from basal organisms (e.g., bacteria and fungi) to coral consumers, also reveals limited bacterial and fungal contributions to coral nutrition. Thus, we advocate that coral nutrition is primarily determined by Symbiodiniaceae translocation and particulate feeding. As these nutritional pathways are highly subject to environmental fluctuations, corals lacking trophic flexibility may suffer more from malnutrition and even population decline under global environmental change.

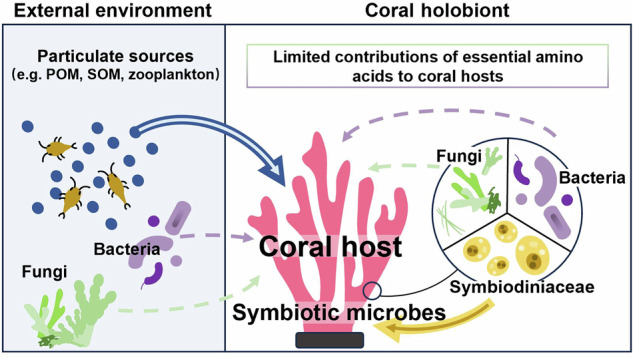

## Abstract (web summary)

Compound-specific stable carbon isotope analysis reveals seasonal changes in the nutritional sources of corals (*Pocillopora damicornis*) as well as limited contributions of microbe-derived essential amino acids to coral nutrition.

## Introduction

Microbes are crucial to marine ecosystems because they play a pivotal role in many ecological processes, such as energy flow and biogeochemical cycles^1^. In particular, some microbes can form a symbiotic relationship with an animal host, known as holobiont^2–4^, and this symbiosis can reshape the resilience and ecological niches of the host^5,6^. Reef-building corals are the representative group of organisms with this relationship, where they host a wide spectrum of microbes, such as microalgae (i.e., Symbiodiniaceae), bacteria and fungi^7^. Since these microbes can contribute to the nutrition and hence growth of corals, they may critically and indirectly influence the community structure and nutrient dynamics of coral reef ecosystems^8,9^.

Physiologically, reef-building corals obtain nutrients largely as photosynthates produced by their algal symbionts through photosynthesis (i.e., autotrophy) to meet their energy demands. Yet, some essential elements, such as nitrogen and phosphorus, can be acquired through consumption of zooplankton and organic matter (i.e., heterotrophy)^10^. It is noteworthy that the autotrophic capacity of corals is highly subject to environmental factors, especially temperature and light availability. For example, thermal stress can break down the coral-algae symbiosis and lead to coral bleaching, thereby lowering the autotrophic capacity of corals^11^. To offset the reduced energy gain via autotrophy, corals may increase their reliance on heterotrophy as a compensatory response^12,13^. Indeed, the heterotrophic capacity varies among species and is closely linked to coral morphology as larger corallites generally support higher heterotrophic capacity and enhance thermal resilience^14^. A recent study showed that some coral species (*Galaxea fascicularis*, *Pachyseris speciosa* and *Pocillopora verrucosa*) can increase their heterotrophic feeding upon exposure to thermal stress and in the subsequent recovery phase^15^. Such flexibility in shifting nutritional modes can act as a crucial strategy of corals to survive in the dynamic marine environment^16,17^.

However, the capacity of trophic flexibility varies among coral species and environmental conditions^18^. For instance, healthy rice coral (*Montipora capitata*) was found to obtain most of its energy from autotrophy, regardless of environmental conditions^19,20^. Yet, it can greatly increase its heterotrophic feeding when bleached^21^. Given the limited nutrient intake through feeding in healthy colonies, the contributions of other microbial partners, such as bacteria and fungi, may become crucial to maintain the energy gain of corals when light is barely available^4,22^. These microbes, which inhabit the tissues, microenvironments (e.g., skeletons) and surrounding biofilms of corals, are capable of synthesizing essential nutrients required by corals, like vitamins, lipids and amino acids^7,23,24^. Therefore, corals could obtain these nutrients either by translocation from their symbionts or by ingestion of microbes via active feeding, such as mucus trapping^7,25^. For example, recent omics analyses showed that adding beneficial bacterial strains (e.g., *Bacillus lehensis* and *Brachybacterium conglomeratum*) to coral aquaria stimulates upregulation of lipid and sterol biosynthesis, which in turn aids coral recover from thermal bleaching^26^, indicating the important role of microbes in coral nutrition and survival. Nevertheless, the limited contributions of coral-associated microbes to coral nutrition in terms of the quantity of amino acids produced were also found^27^. This can be exemplified by microbial metagenome analyses that bacteria associated with mustard hill coral (*Porites astreoides*) are mostly heterotrophs with a limited capacity for carbon and nitrogen fixation^28^. To resolve this inconsistency, more studies are needed to explore the extent to which coral-associated microbes contribute to coral nutrition, which can shed light on the capacity of corals to maintain energy homeostasis via trophic flexibility—a possible mechanism enabling corals to adjust to rapidly changing environments.

To assess the microbial contributions to coral nutrition, compound-specific carbon isotope (δ^13^C) analysis of amino acids (CSIA-AA) can be used as an advanced analytical tool^29,30^. Since corals, like other animal consumers, cannot synthesize certain proteinogenic amino acids^7,23^, known as essential amino acids (EAAs), they must obtain these compounds from basal organism groups (e.g., microalgae, bacteria and fungi) that serve as the fundamental building blocks of proteins. As these basal organisms synthesize EAAs (i.e., basal resources) via distinct metabolic pathways, they have unique intermolecular δ^13^C relationships among EAAs^31,32^, creating a taxon-specific ‘fingerprint’^33,34^. When animals consume these EAAs, their δ^13^C pattern or fingerprint is retained with minimal alteration through trophic transfer^29^, which helps identify microbial EAA origins scaling from individuals to ecosystems^35,36^. Indeed, recent works demonstrated that CSIA-AA can reliably trace EAA provisioning in basal resources, enabling the identification of nutritional sources for corals^20,37,38^ and other marine organisms^6,39^. In contrast, non-essential amino acids (NEAAs) can be synthesized de novo by most metazoans and hence their δ^13^C values reflect their precursor carbon pools and metabolic routing, which respond to health status and environmental conditions^30,31,40^. Therefore, δ^13^C_NEAA_ values integrate dietary inputs, internal routing, physiological state and environmental background, complicating their use as straightforward source tracers yet providing valuable insights into individual metabolic dynamics and spatiotemporal use of resources.

While trophic flexibility underpins coral resilience to environmental stress^14,21^, most reef restoration and conservation plans overlook corals’ heterotrophic capacity even though global environmental change drives widespread coral declines^9,41^. Here, we aim to evaluate the contributions of potential sources (Symbiodiniaceae, bacteria, fungi and heterotrophic food sources) to coral nutrition across seasons (cool and warm) by measuring the δ^13^C values of 10 individual AAs from the in situ heterotrophic food sources (particulate organic matter (POM), sedimentary organic matter (SOM) and zooplankton), coral hosts and Symbiodiniaceae. A widespread mixotrophic coral *Pocillopora damicornis* was selected as a model species, which is known to host about 400 different bacterial strains in addition to Symbiodiniaceae^23^. Given the high abundance of bacteria and fungi in the microbiome of corals^7,42^, we hypothesize that (1) the δ^13^C_EAA_ patterns of coral hosts would overlap with the δ^13^C_EAA_ fingerprints of bacteria and/or fungi if both autotrophic and heterotrophic capacity of corals are constrained (e.g., reduced food availability or algal symbiont density), and (2) the δ^13^C_EAA_ patterns of *P. damicornis* coral host would shift from Symbiodiniaceae fingerprint to heterotrophic food source fingerprint across seasons, which reflects the ability of this coral species to switch nutritional modes in response to environmental change. Furthermore, we compiled the published δ^13^C_EAA_ data of other coral species (e.g., *Pocillopora meandrina*^38^, *M. capitata*^20^ and *Isidella* sp.^43^) to estimate the nutritional contributions of bacteria and fungi to different coral species. Taken together, this study comprehensively evaluates the role of bacteria and fungi in coral nutrition, which can provide critical insights into the resilience of corals to nutrient-limited conditions as well as their persistence in ever-changing marine ecosystems.

## Results

### Isotopic signals in corals, Symbiodiniaceae and heterotrophic sources

**Fig. 1:**
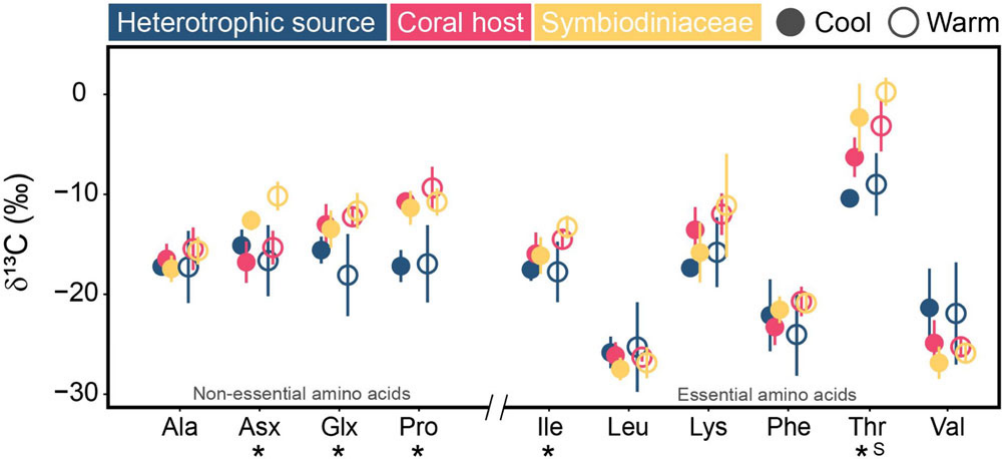
Measured δ^13^C values of various amino acids. Comparisons of the δ^13^C values of four non-essential (Ala, alanine; Asx, aspartic acid/asparagine; Glx, glutamic acid/glutamine; Pro, proline) and six essential (Ile, isoleucine; Leu, leucine; Lys, lysine; Phe, phenylalanine; Thr, threonine; Val, valine) amino acids in the putative heterotrophic source, coral host (*Pocillopora damicornis*) and Symbiodiniaceae fractions in the cool and warm seasons. Data are expressed as mean ±:S.D. (*n* = 3 and *n* = 5 biologically independent samples for heterotrophic source and *P. damicornis*, respectively). Significant differences (*p* < 0.05) within each amino acid between sample fractions or seasons are indicated by (*) and (S), respectively.

**Fig. 2:**
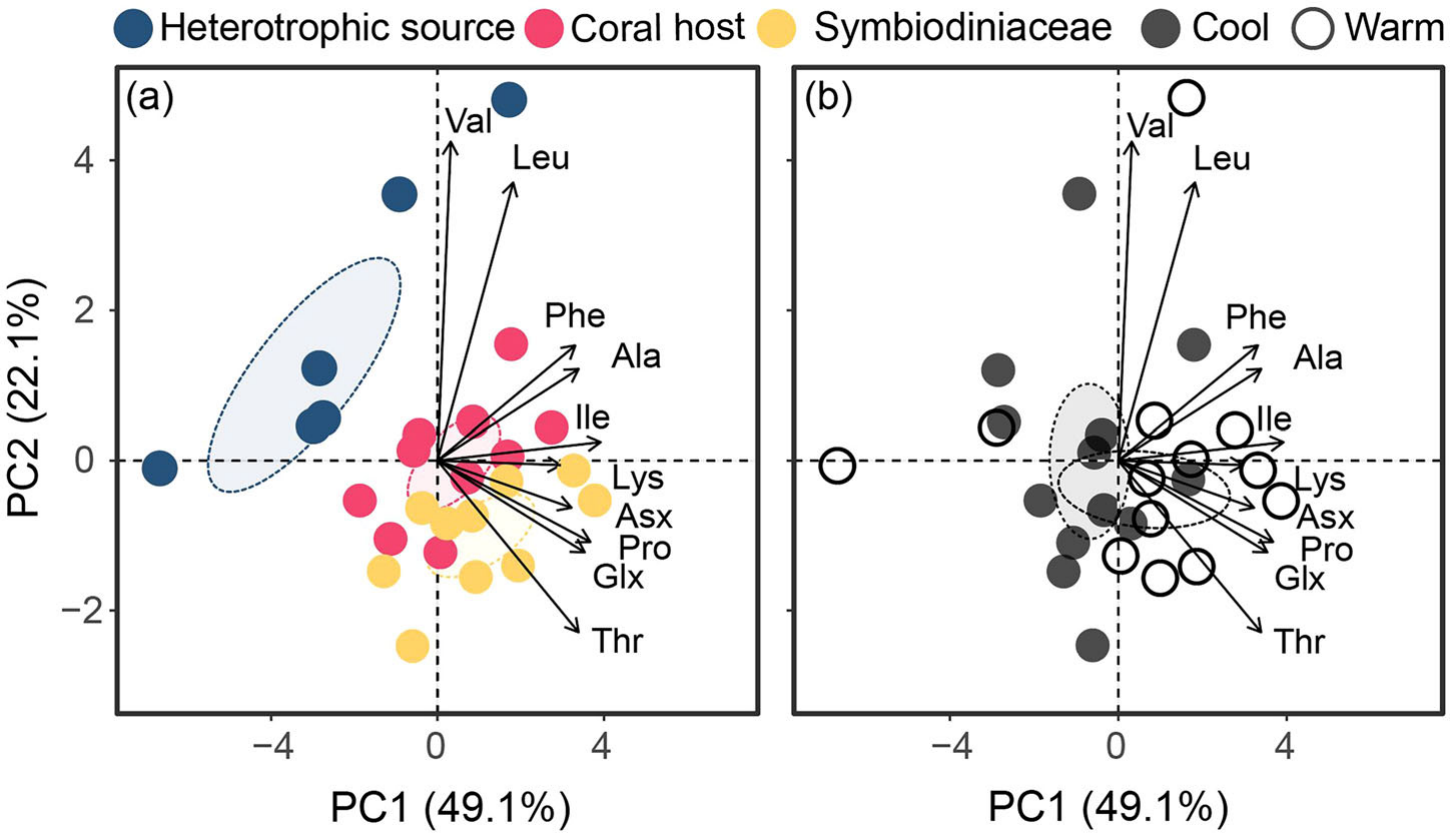
Multivariate analyses of measured δ^13^C_AA_ values. Principal component analysis of 10 measured δ^13^C_AA_ values to evaluate the effects of **a** sample fractions (*p* < 0.01) and **b** seasonality (*p* = 0.04). The ellipses represent the 95% confidence intervals of the group averages, while the arrows represent significant correlations for the first two principal components.

On average, the measured δ^13^C_AA_ values ranged from −25.4 ± 1.8‰ for Leu to −4.1 ± 4.2‰ for Thr (Fig. 1, Supplementary Table 1). The variation in measured δ^13^C values between individual amino acids was generally larger than those within the same amino acid among the three sample pools (Fig. 1). Yet, significant differences in measured δ^13^C values were observed among the three sample pools for three NEAAs and two EAAs (Fig. 1, Supplementary Table 2). Specifically, for the NEAAs, the measured δ^13^C values of Pro (*p* < 0.01) and Glx (*p* < 0.01) were significantly higher in both coral hosts and Symbiodiniaceae than heterotrophic sources, while the δ^13^C_Asx_ values were the greatest in Symbiodiniaceae relative to coral hosts and heterotrophic sources (*p* < 0.01). For the EAAs, the host values usually ranged between two nutrient source values, with measured δ^13^C values significantly lower in Ile (*p* < 0.01) and Thr (*p* < 0.01) for heterotrophic sources. Only Thr had seasonal differences (*p* = 0.05), with higher δ^13^C values in the warm season than the cool season (Supplementary Table 2). Principal component analysis (PCA) and permutational analysis of variance (PERMANOVA) results showed significant effects of the pooled sample fractions (*p* < 0.01) and seasonality (*p* = 0.04; Fig. 2), but not their interaction (*p* = 0.43), on the overall pattern of measured δ^13^C_AA_ values (Supplementary Tables 3 and 4).

### Estimation of the relative importance of basal resources to corals

**Fig. 3:**
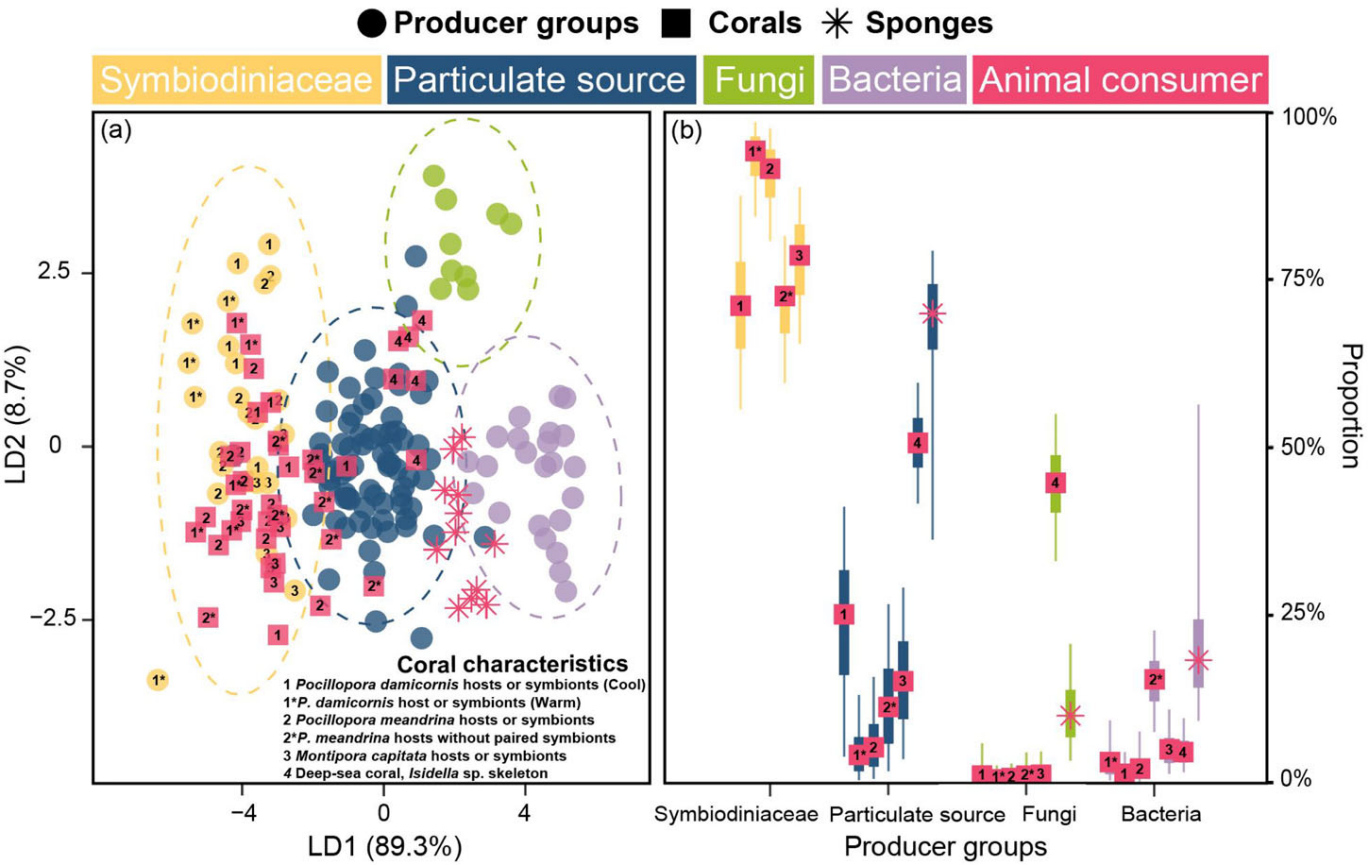
Estimation of basal resources for consumers. **a** Linear discriminant analysis based on the six mean-centered δ^13^C_EAA_ values (Ile, Leu, Lys, Phe, Thr and Val) for four basal resources groups (training data: Symbiodiniaceae, fungi, bacteria and particulate source) with corals and sponges. The ellipses represent the 95% confidence intervals of each source. The colors indicate taxonomic groups. The solid circles represent basal resources, while squares and stars represent corals and sponges, respectively. **b** Posterior probabilities of the proportionate contribution of each basal resources groups as estimated by MixSIAR for corals (pink square) and sponges (pink star). The squares and stars at the center of each box denote posterior median, with box and whisker representing 50% credible interval and 90% credible interval, respectively. The numbers indicate the species name and information of coral samples included in the analysis. The details of this dataset are shown in the Supplementary Data 1.

The linear discriminant analysis (LDA) results of the mean-centered δ^13^C_EAA_ values from the overall samples, which included Symbiodiniaceae (*n* = 27), particulate sources (*n* = 75), fungi (*n* = 9) and bacteria (*n* = 23) as basal organism groups, showed that these four groups were well separated from each other, with a high reclassification rate (95.5%) within the designated groups (Fig. 3; Supplementary Table 5). As suggested by the first two LDA factors, the mean-centered δ^13^C_EAA_ pattern for the corals and sponges was distributed among the four groups. The LDA classified 88.5% (23 of 26 samples; with paired Symbiodiniaceae data as the source) and 44.5% (4 of 9 samples; without paired Symbiodiniaceae data as the source) of reef-building coral hosts into the Symbiodiniaceae group, while 11.5% (3 of 26 samples; with paired Symbiodiniaceae data as the source) and 55.5% (5 of 9 samples; without paired Symbiodiniaceae data as the source) of reef-building coral hosts, 100% of deep-sea corals (6 samples) and 66.7% of sponges (8 of 12 samples) were assigned as particulate sources (Fig. 3a). In addition, 33.4% of sponges (4 of 12 samples) were assigned as bacteria, whereas no animal samples were classified into the fungi group (Fig. 3a).

Bayesian mixing model results revealed nutritional contributions of basal resources to corals and sponges, similar to the classification based on LDA (Fig. 3b). Specifically, Symbiodiniaceae contributed a range of 70.7–93.8%, while particulate sources accounted for an estimated range of 4.8–24.8% in reef-building coral hosts (Fig. 3b). Fungus-derived resources had the lowest contribution consistently, ranging from 0.3 to 0.9%, while bacterial contributions were generally low, ranging from 1.1 to 4.6% for most coral hosts (Fig. 3b). However, some corals without paired Symbiodiniaceae showed a higher bacterial contribution of 15.1% (Fig. 3b). For deep-sea corals and sponges, MixSIAR models estimated that particulate sources were the primary contributors, accounting for more than 50% in both groups. However, the relative contributions of bacterial and fungal EAAs varied between deep-sea corals (4.5% and 44.6%, respectively) and sponges (18.7% and 9.0%, respectively).

### Estimation of trophic plasticity of the regional coral samples

**Fig. 4:**
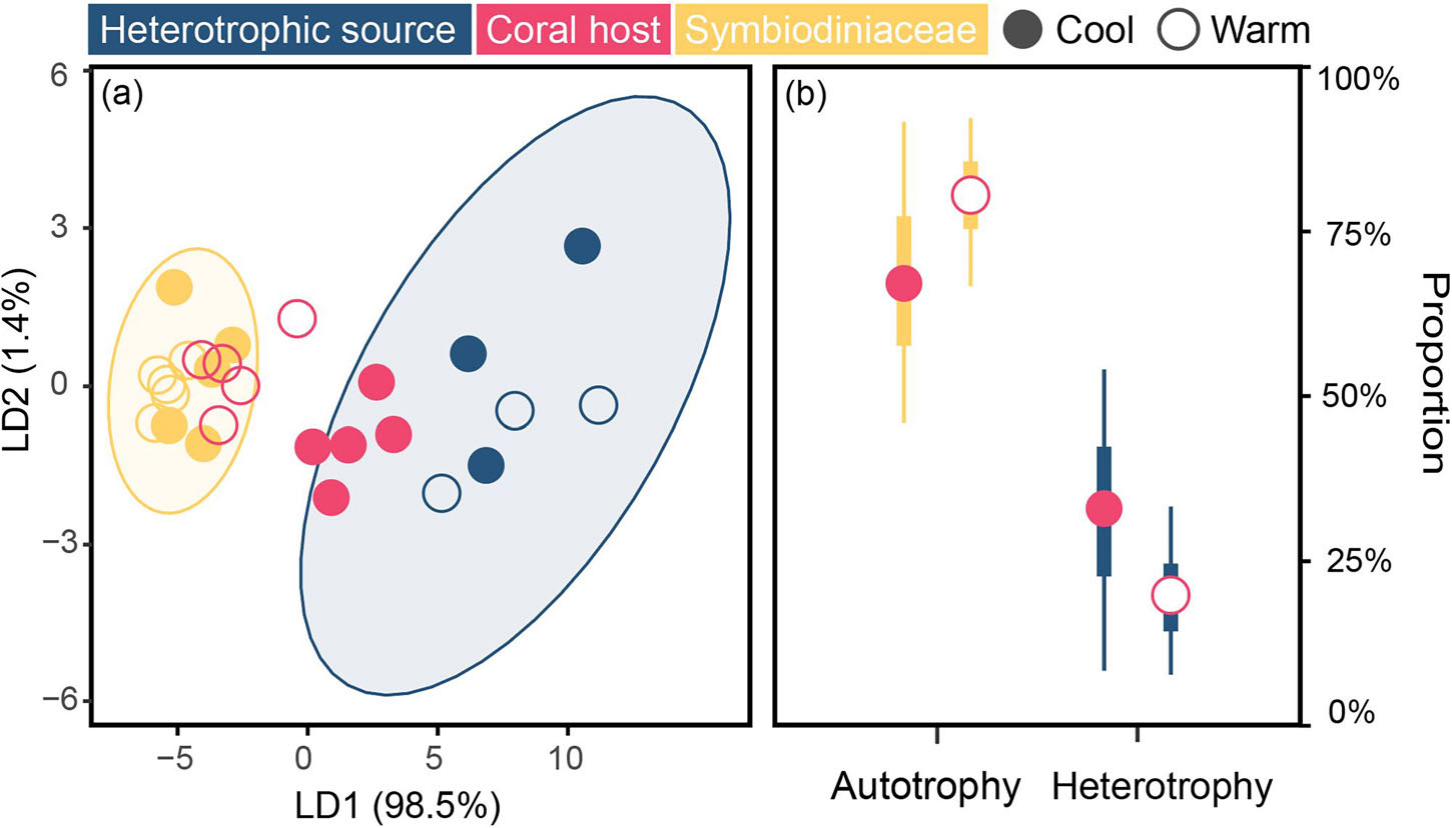
Estimation of trophic plasticity in the regional corals. **a** Linear discriminant analysis based on six mean-centered δ^13^C_EAA_ values (Ile, Leu, Lys, Phe, Thr and Val) from the regional samples. **b** Proportional contribution of autotrophy and heterotrophy to regional corals across seasons based on MixSIAR. The circles, box and whisker denote posterior medians, 50% credible interval and 90% credible interval, respectively.

As the first two factors of the LDA (Supplementary Table 6), the mean-centered δ^13^C_EAA_ values of the heterotrophic sources and Symbiodiniaceae were clustered separately. The regional coral hosts were distributed between the two basal resource groups. The LDA assigned one cool-season and five warm-season coral hosts to the Symbiodiniaceae group, whereas the remaining four cool-season coral hosts were classified into the heterotrophic source group (Fig. 4a), implying enhanced heterotrophic reliance under cool conditions. This trend was reflected by regional MixSIAR results, with estimated median heterotrophic contributions of 32.9% in the cool season and 19.5% in the warm season **(**Fig. 4b**)**. This seasonal shift in heterotrophy was even more pronounced in overall mixing models, with a 21.0% declined in heterotrophy reliance from the cool season to the warm season **(**Fig. 3b).

## Discussion

Bacteria and fungi are recognized for their ability to synthesize essential nutrients for coral hosts, possibly playing a crucial role in coral nutrition when food density or light availability is low. By analyzing isotopic profiles of EAAs, however, we found that bacteria and fungi did not contribute substantially to coral nutrition, even when nutrient translocation from symbiotic algae was limited. These findings highlight the fundamental role of external particulate food sources (e.g., zooplankton) rather than coral-associated microbes or microbial loops (e.g., nutrients derived from bacteria and fungi) in supplementing coral nutrition, which can support the resilience of corals to environmental change.

### Contributions of bacteria and fungi to coral nutrition

Symbiodiniaceae are well known for supplying corals with carbohydrates via photosynthesis, but recent studies suggest bacteria and fungi may also play a role due to their high diversity within coral holobionts^22,42,44,45^. In fact, these microbes can synthesize essential nutrients (e.g., EAAs and B-vitamins) and provide crucial biochemical pathways (e.g., nitrogen fixation), which cannot be found in coral hosts^7,23,46^. Yet, studies on how much of these nutrients are actually derived from bacteria and fungi are scant. Here, we revealed limited nutritional contributions of bacteria and fungi as evidenced by a lack of overlap between their δ^13^C_EAA_ fingerprints and the δ^13^C_EAA_ patterns of reef-building corals. However, ~40% of sponge individuals in the analysis (*Xestospongia muta*, *Agelas tubulata* and *Plakortis angulospiculatu*) relied heavily on bacterial nutrition, indicated by their overlapping δ^13^C_EAA_ patterns with bacteria and estimation of proportional contribution by MixSIAR (Fig. 3). This aligns with previous findings that sponges largely utilize organic matter derived from bacteria, which synthesize and translocate essential nutrients to hosts^3,6^. In coral holobionts, bacteria and fungi likely play a secondary role in nutrition by consuming excess nutrients produced by Symbiodiniaceae or coral metabolic waste^28^, rather than serve as primary nutrient producers. This difference may be due to the fact that the sponges included in our study are classified as high-microbial-abundance sponges and lack algal symbionts as primary producers, meaning that symbiotic bacteria are indispensable for synthesizing these essential compounds^6,44^. In short, our findings suggest that microbes may aid nutrient cycling within coral holobionts, but their direct nutritional contributions to corals are limited compared to those of Symbiodiniaceae.

Nevertheless, the contributions of bacteria and fungi to coral nutrition may vary among species, as corals have evolved diverse nutrient acquisition strategies^14,16,45^. For instance, the strength of symbiotic relationships between corals and Symbiodiniaceae can determine coral nutrition^13,47^, thereby influencing the reliance of corals on other potential food sources. In this study, the basal resources of reef-building corals only switched between particulate food sources and Symbiodiniaceae-derived nutrients, while the contributions of bacteria and fungi appeared to be negligible (Fig. 3a). Specifically, corals without Symbiodiniaceae (e.g., *Isidella* sp. from deep sea and *P. meandrina* from shallow waters) relied heavily on particulate food sources, as indicated by their δ^13^C_EAA_ patterns. While the δ^13^C_EAA_ patterns of certain deep-sea corals can overlap with δ^13^C_EAA_ fingerprints of fungi, possibly due to the higher fungal diversity and amino acid production via fungal degradation^33,48^, these corals still depend largely on particulate food sources. In *P. meandrina* hosts without Symbiodiniaceae, MixSIAR modeling indicates that bacteria supply ~15.1% of the host’s EAAs, a contribution comparable to particulate feeding (Fig. 3b). In addition, corals with branching or plating skeletal morphology (e.g., *P. damicornis*, *P. meandrina* and *M. capitata* in this study) often depend more heavily on nutrients derived from Symbiodiniaceae due to their relatively small-polyp sizes^49^. In contrast, large-polyp corals (e.g., *Fimbriaphyllia ancora*), which have abundant and diverse bacteria (e.g., Gammaproteobacteria) growing around their mouth^50^, can readily take up dissolved EAAs by consuming these microorganisms. These corals were also shown to have lower absorption rates of dissolved EAAs from external water than small-polyp corals^51^, suggesting that bacterial and fungal EAAs play a more important role in the nutrition of large-polyp corals. Furthermore, while distinct biosynthetic pathways of basal organisms shape their δ^13^C_EAA_ fingerprints, most bacterial and fungal δ^13^C_EAA_ data come from terrestrial or laboratory cultures^30,33,52^. Yet, aquatic microbial signatures remain largely uncharacterized, and their oversight may lead us to underestimate microbial contributions to consumers. This is particularly true in view of the within‑group variability in δ^13^C_EAA_ fingerprints, such as that observed in marine algae^53^. Future studies should therefore explore the δ^13^C_EAA_ fingerprints of aquatic bacteria and fungi to ascertain the nutritional role of microbial symbionts in large-polyp, stress-tolerant corals.

### Seasonal variation in the contributions of different EAA sources to coral nutrition

Marine bacteria, particularly those associated with corals, are sensitive to environmental change, such as temperature fluctuations. Thus, their diversity and abundance in coral reefs often vary between seasons^54^, possibly affecting their nutritional contributions to corals. For instance, in subtropical coral reefs like those in the South China Sea, elevated sea surface temperature in summer (~31 °C; close to the bleaching threshold of corals) greatly reduced the abundance of *Endozoicomonas* sp. in coral *P. damicornis* by half in terms of operational taxonomic units^55^, while the dominant phylum Proteobacteria in *Acropora pruinosa* decreased by ~60%^56^. Given that these bacteria are responsible for nutrient cycling and synthesis of essential nutrients for corals^7,57^, it is reasonable to infer that corals need to depend more on bacteria-derived nutrients in cooler seasons (e.g., winter) when the photosynthetic activity of Symbiodiniaceae is less intense and the abundance of nutrient-producing bacteria is higher. However, the consistent measured δ^13^C values for Ile, Val and Leu and low proportional contribution estimated by MixSIAR (1.1 to 2.7%) observed in coral hosts across seasons suggest the limited bacterial and fungal contributions to coral nutrition. Indeed, host δ^13^C values can be subject to the seasonal variation in the incorporation of bacterial EAAs (e.g., decreased values for Ile and Val or increased values for Leu, due to the distinct carbon isotope fractionation by bacterial enzymes, such as acetohydroxy acid synthetase)^39,58^.

Instead, we observed a shift in the *P. damicornis* δ^13^C_EAA_ patterns on regional scale from Symbiodiniaceae signatures to those resembling particulate food sources in overall estimation. This observation aligns with previous ones that *Pocillopora* corals can exhibit trophic flexibility^38^, switching from autotrophy in summer to heterotrophy in winter^47^, to obtain essential nutrients when autotrophy becomes less efficient^12,13,15^. Among NEAAs, Glx and Pro in the coral host and Symbiodiniaceae shared nearly identical and elevated δ^13^C values relative to particulate food sources. This convergence likely reflects de novo synthesis from a shared α‑ketoglutarate pool via transamination (i.e., involving nitrogen atoms), a reaction that imparts negligible ^13^C fractionation and utilizes the same carbon pools^30,31^. To unravel the nitrogen flow underlying these patterns, future studies can incorporate compound-specific nitrogen isotope measurements. In contrast, δ^13^C values of Asx in Symbiodiniaceae were higher than those in coral host and its food by 5‰, which was far beyond typical trophic enrichment. This suggests the limited exchange of Asx between the coral host and Symbiodiniaceae as well as the large isotope effects during Asx synthesis via carboxylation/decarboxylation, consistent with the capacity of corals for substantial de novo aspartate production^31^.

It is noteworthy that corals are also capable of actively feeding on bacteria, ingesting more than 10^7^ bacterial cells per cm^2^ per hour^10,59^. Feeding on bacteria can help corals acquire EAAs^25^, especially when heterotrophic activity is enhanced. In our case, however, the contributions of bacteria and fungi to EAAs remained low even in winter, when *P. damicornis* relied more on heterotrophy^47^ (Fig. 3). This observation may result from the differences in the nutritional quality among nutrient sources. Specifically, particulate food sources (e.g., zooplankton) are richer in amino acids, lipids and other nutrients than bacteria and fungi^25^, possibly leading to a greater assimilation of EAAs from particulate sources than from bacterial or fungal sources. In addition, bacteria and fungi often lack essential sterols^5^, which are critical for coral tissue function and energy metabolism. Such absence of sterols may restrict efficient assimilation of EAAs from bacterial and fungal sources through co-limitation effects that the scarcity of one nutrient impairs the utilization of another^60^. Overall, our findings suggest that *P. damicornis* can change its trophic strategy to obtain nutrients in response to seasonal environmental change. Yet, bacteria and fungi appear to have limited contributions to *P. damicornis* nutrition even when photosynthates provided by Symbiodiniaceae decline.

### Potential shifts in coral nutrition in response to global environmental change

Global warming has been suggested to disrupt coral-Symbiodiniaceae symbiosis^61^ or even change symbiotic relationship from mutualism to parasitism^62^, which could lower the nutritional contributions of Symbiodiniaceae to corals. Under these circumstances, heterotrophic feeding (e.g., capturing high-quality zooplankton) would become more important for corals to meet their energy demands^14^. However, zooplankton populations are projected to decline by more than 10% by the end of this century as a result of climate change^63^, implying that corals may occasionally suffer from malnutrition in this scenario. Although bacteria and fungi could supplement essential nutrients to corals^8,57^, the current findings suggest that their direct contributions to coral nutrition is scant, even when corals can rely more on heterotrophic feeding (e.g., during recovery from bleaching events or seasonal shifts). Climate change is also predicted to lower the abundance of beneficial bacteria^55,64^, further challenging the survival of corals under ocean warming.

Despite the lack of evidence in this study showing direct EAA contributions of bacteria and fungi to corals, they may indirectly help corals assimilate AAs. Metagenomic analyses have identified numerous genes in coral-associated bacteria and fungi essential for nutrient cycling^28,57^, particularly nitrogen fixation that converts nitrogen gas into biologically available ammonium^6,46^. This process, which results in signified nitrogen isotope values close to 0‰^65^, was observed in both the symbiotic algae of corals and their reef environments^18,47^. Under ocean warming, nitrogen-fixing bacteria may become more abundant^66^, suggesting that algal biosynthesis of EAAs for the coral host can be boosted to increase the resilience of corals. The increased ammonium availability could also enhance the coral host’s capacity for de novo synthesis of NEAAs^67^. Many microalgae, including dinoflagellates, rely on bacteria-produced B vitamins (e.g., cobalamin), which can neither be synthesized de novo by themselves nor their coral hosts. As a result, algal autotrophic capacity becomes limited and the metabolic support that reef‑building corals require is compromised^23,68^. Nevertheless, a recent long-term study on coral microbiomes exposed to warming and acidification for 22 months revealed the ability of corals to adjust their microbial communities intra- and inter-specifically^69^. This finding suggests that corals may modify their microbial composition to enhance nutrient cycling when the availability of other food sources is reduced^2,18^. However, the adaptive value of this change is likely species-specific and depends on the inherent biological traits of corals and the composition of their associated microbial communities. Therefore, further studies are required to delve into how global environmental change alters the interactions between coral hosts and their associated microbial assemblages so that the resilience of corals to future climate scenarios can be estimated.

**Fig. 5:**
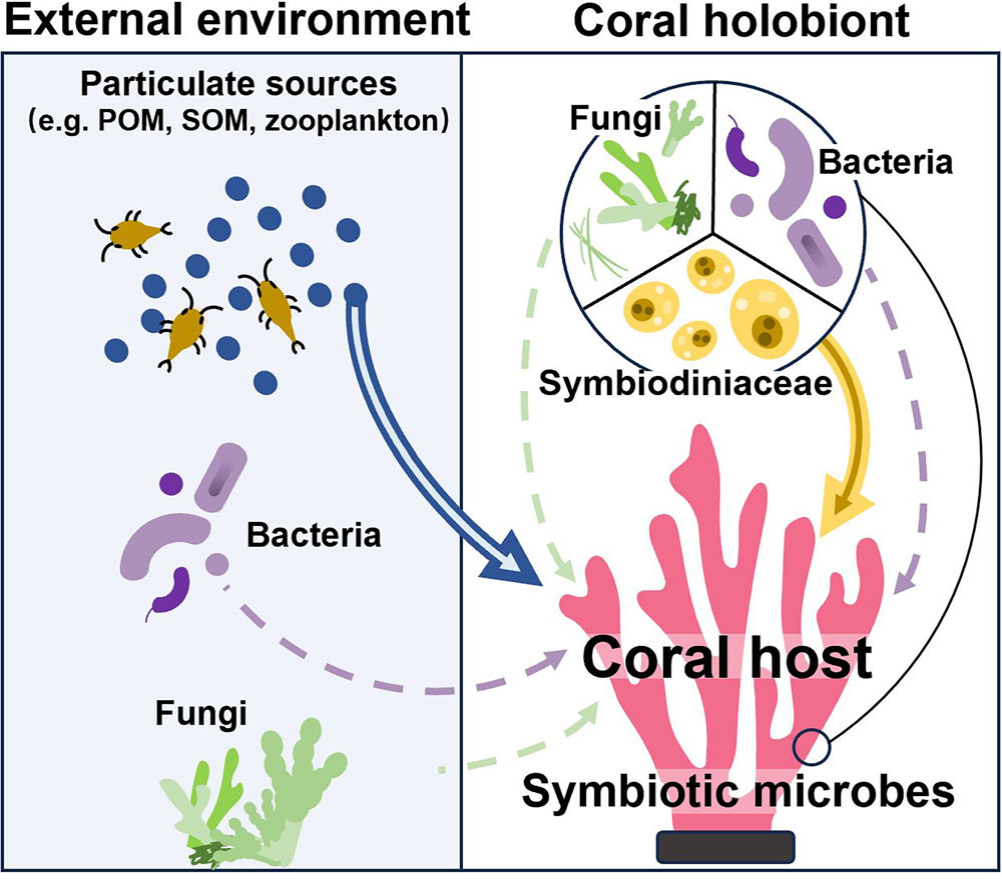
A conceptual diagram illustrating the functional roles of various groups of primary producers in coral nutrition. Compared to Symbiodiniaceae, bacteria and fungi were found to have limited nutritional contributions to coral hosts. The arrows indicate the flux of essential nutrient (i.e., essential amino acids) provision. The solid arrows represent the major pathways of nutritional contributions to coral hosts, depending on the abundance of sources, whereas the dotted arrows indicate minor or no nutritional contribution to coral hosts.

In conclusion, corals are sensitive to environmental fluctuations and likely threatened by global environmental change that may reduce nourishment by their symbiotic algae or undermine their capacity to capture food particles. While coral-associated microbes may help supplement their coral hosts with essential nutrients under such conditions, their actual nutritional contributions remain uncertain. Irrespective of coral species and their autotrophic capacity, this study revealed limited contributions of bacteria and fungi to coral nutrition across seasons based on the profiles of EAAs (Fig. 5). Instead, some corals relied more on external particulate food sources to meet their nutritional needs when their autotrophic capacity was reduced. These findings challenge the notion about the important role of coral-associated microbes in coral nutrition, and corroborate that Symbiodiniaceae and particulate food sources are indispensable nutrient sources for corals. This study also highlights the capacity of corals to shift their nutritional modes between autotrophy and heterotrophy in response to seasonal change in environmental conditions, which can allow them to meet their nutritional requirements throughout the year and even adapt to global environmental change. Future research on the trophic flexibility and adaptability of different types of coral species in response to environmental stress is recommended using δ^13^C_EAA_ fingerprints, which can help predict the persistence of corals and their ecological contributions to coral reefs in the changing ocean.

## Methods

### Study area and sample collection

Sanya Bay, which is located in the southern coast of Hainan Island, China, was chosen as the study area. The coral coverage in this region has declined from over 60% to about 20% since the late 19th century, mainly due to anthropogenic activities^41^. In this study, samples were collected from the Houhai fringe reef located in the eastern Sanya (18.276°N, 109.726°E). Currently, this reef is designated as a refuge for corals due to the cooler upwelling in summer^70^. Over 50 coral species (~50% benthic cover) have been documented in this reef, with *P. damicornis* as a dominant species^71^.

Between November 2019 and June 2020, seawater temperature increased from 22 °C to 28 °C in the Houhai fringe reef, while chlorophyll concentration (Chl *a*) decreased from ~4 to 2 mg m^-3^, subject to the abundance of copepods – the primary zooplankton group^72,73^. To capture the main heterotrophic sources available to corals, we collected three putative food sources (*n* = 3 per season per source, but source triplicates were pooled into a single sample) at a depth of 2 m: (1) SOM samples scraped from the upper surface of the reef sediments, (2) POM samples obtained by filtering 5 L of subsurface seawater onto a pre-combusted Whatman GF/F filter (0.7 μm), and (3) zooplankton samples (mainly copepods) collected using a plankton net (mesh size: 163 μm)^38,73^. At the same depth, five healthy colonies of *P. damicornis* were sampled per season by SCUBA divers using a hammer and chisel. Specifically, we focused on the upper 1.5 cm of several branches for isotope analysis. With a local linear growth rate of *P. damicornis* about 6 cm per year and a tissue turnover time about 3 months^73,74^,the isotopic values in coral branches were analysed in November 2019 and June 2020, corresponding to late autumn–early winter (cool season) and late spring–early summer (warm season), respectively. All samples were flash-frozen in liquid nitrogen and stored at –20 °C until analysis.

### Sample treatment and isotopic analysis

Coral tissues, including the hosts and Symbiodiniaceae, were removed by an air-brush system with deionized water, collected in Corning tubes and finally homogenized on an ice bath, following an established centrifugation protocol^47^. In brief, the homogenate was centrifuged at 1500 × *g* at 4 °C for 10 min, resulting in the supernatant (mainly host tissue) and pellets (mainly Symbiodiniaceae). The supernatant, containing the host fraction, was centrifuged for four rounds (3000 × *g* at 4 °C for 10 min) to remove the remaining Symbiodiniaceae (i.e., pellets discarded). The initial Symbiodiniaceae pellets were resuspended in deionized water and centrifuged for five rounds (500 × *g* at 4 °C for 10 min) to purify the Symbiodiniaceae fraction and eliminate the coral tissue remnants (i.e., supernatant discarded). All samples were lyophilized before the isotopic analysis.

All samples were analysed for their amino acid δ^13^C (δ^13^C_AA_) values, following a previous protocol^75,76^. The lyophilized samples (~1 mg) with an internal standard added (norleucine, Nor; Sigma-Aldrich) were hydrolysed in 6 N HCl at 110 °C for 20 h. The samples were then purified with cation exchange chromatography (Dowex 50WX8 hydrogen form; Sigma-Aldrich) and derivatized based on a modified method^77^. Specifically, the purified samples were esterified by 20% acetyl chloride in 2-propanol at 110 °C for 1 h, followed by acylation with 25% trifluoroacetic anhydride in dichloromethane at 110 °C for 15 min. To calibrate the carbon derived^78^, external standards of 12 mixed amino acids (with known δ^13^C values) were analysed in parallel: alanine (Ala), glycine (Gly), threonine (Thr), valine (Val), leucine (Leu), isoleucine (Ile), proline (Pro), asparagine/aspartic acid (Asx), glutamine/glutamic acid (Glx), phenylalanine (Phe), tyrosine (Tyr) and lysine (Lys). The amino acids in the prepared samples and external standards were separated using a gas chromatograph column (Agilent Technologies, 60 m × 0.25 mm × 0.25 μm, DB-5MS; Supplementary Fig. 1), and combusted into CO_2_ (GC Isolink interface). The δ^13^C values of individual peaks were measured by IRMS (Thermo Delta V Advantage). Instrument performance and drift were monitored using the mixed *n*-alkane isotope standard B5 (Indiana University), while calibration of AA mix standards and samples was performed using AA standards with known δ^13^C values. Both external standards (i.e., derivatized mixed AAs and B5) were analysed before and after each set of three sample injections. Triplicate measurements were performed for each sample (see Supplementary Table 1 for the δ^13^C values of individual amino acids in each sample) and isotopic values were reported in δ-notation relative to V-PDB. The average standard deviation for the internal standard (Nor), which involved 2–3 injections per sample (*n* = 26), was 0.28‰. A total of 12 δ^13^C of amino acids (δ^13^C_AA_) were analysed, but we only reported 10 of them (Ala, Thr, Val, Leu, Ile, Pro, Asx, Glx, Phe and Lys) because Tyr was not detected in some samples, whereas Gly had technical-replicate standard deviations greater than 1‰. The 10 amino acids tested in this study were classified into essential (EAA; Ile, Leu, Lys, Phe, Thr and Val) and non-essential (NEAA; Ala, Asx, Glx and Pro) amino acids based on previous studies^20,38,40^.

### Statistical analyses

All statistical analyses were conducted in R version 4.0.5. Since POM is consumed by zooplankton and SOM is mainly derived from benthic microalgae^38^, we assumed that POM, SOM and zooplankton would have similar overall δ^13^C_EAA_ patterns, as reported in Fox et al.^38^. As our main objective is to assess the relative contributions of endogenous or allochthonous food to coral nutrition, we combined and denoted these sources as “heterotrophic sources” to reflect the natural variation in their availability to corals. The overall measured δ^13^C_AA_ values of the samples were assessed across seasons (cool and warm) and sample fractions (i.e., coral hosts, Symbiodiniaceae and heterotrophic sources) using PERMANOVA. PCA was performed to visualize the relationship among sample fractions across seasons using the *FactoMineR* package^79^. To test the fractional and seasonal differences in the individual δ^13^C_AA_ values, two-way ANOVA followed by pairwise comparison was conducted with seasons and sample fractions as the fixed factors. Levene’s test and Shapiro-Wilk’s test were used to verify the assumptions of homoscedasticity and normality, respectively.

We used two models to trace the origin of EAAs in corals. First, LDA was applied to predict the group members (i.e., different basal resource groups) of coral hosts. Specifically, LDA (R: *MASS* package) was used to construct axes that maximize the separation between basal resource groups using the mean-centered carbon isotope values of each EAA (mean-centered δ^13^C_EAA_ = targeted δ^13^C_EAA_ – mean δ^13^C value for 6 EAAs; Ile, Leu, Lys, Phe, Thr and Val), following the standard δ^13^C_EAA_ fingerprint approach^30,33,80^. The measured δ^13^C_EAA_ values were influenced by various factors (e.g., locations and environmental parameters), but the mean-centring process effectively minimizes these effects while preserving the relative isotope fractionation differences among basal organisms. To create a robust δ^13^C_EAA_ fingerprint for basal resource groups, a total of 118 reference basal organisms were compiled from published studies that focus on the same type of 6 EAAs^20,33,34,38,43,52^ including Symbiodiniaceae (*n* = 17; from laboratory cultures and Palmyra), microalgae (*n* = 27; from laboratory cultures), zooplankton, POM as well as sinking particles (*n* = 42; from Hawaii, Palmyra and North Pacific Ocean; >0.7 μm), fungi (*n* = 9; from terrestrial cultures) and bacteria (*n* = 23; from terrestrial cultures), constituting the training data. Meanwhile, zooplankton, POM, sinking particles and microalgae reference samples were combined into a single basal resource group referred to as “particulate sources” based on the assumption described above^20,38^. The mean-centered δ^13^C_EAA_ data from Symbiodiniaceae (*n* = 10) and heterotrophic sources (*n* = 6) in the present study were also incorporated into the model for validation. Our test dataset for corals, comprising 41 consumer samples^20,38,43^ with both experimental (reef-building corals in Hawaii, *M. capitata* under different feeding regimes, *n* = 6) and field corals (reef-building corals in Palmyra, *P. meandrina* from 4 different sites, *n* = 10 and *n* = 9 for hosts with and without (i.e., suggested lower autotrophic capacity) paired Symbiodiniaceae data as sources, respectively; deep-sea corals in North Pacific Ocean, *Isidella* sp. skeleton representing different years, *n* = 6; reef-building corals in this study, *P. damicornis* across seasons, *n* = 10), which can be used to predict their inclusion within the four established basal resource groups (i.e., Symbiodiniaceae, particulate sources, fungi and bacteria) through LDA and thus evaluate the EAA contributions of fungi and bacteria to corals across species and on a larger geographic scale. Furthermore, the same predictive model was applied for an additional symbiotic animal group (i.e., sponges) from previous studies to verify whether LDA can trace the EAA contributions from microbes^6^. The original δ^13^C_EAA_ data compiled with detailed information are shown in the Supplementary Data 1. We also performed LDA for mean-centered δ^13^C_EAA_ values of our regional samples (see comparison of different methods in Supplementary Fig. 2), with heterotrophic sources (*n* = 6) and Symbiodiniaceae (*n* = 10) included in the training dataset to predict the inclusion of coral hosts collected from different seasons and to assess the trophic plasticity of the corals. If seasonal shifts in the major nutritional mode of corals occurred, the host δ^13^C_EAA_ patterns in the regional LDA would be changed.

Second, Bayesian isotope mixing model (R: *MixSIAR* package) was conducted to estimate the percentage contributions of basal resource groups to consumers^30,81^. For the overall model, we used the six mean-centered δ^13^C_EAA_ values, while for the regional model, we used the six measured δ^13^C_EAA_ values (number of chains = 3; chain length = 100,000; burn in = 50,000; and thin = 50). In addition, Symbiodiniaceae was excluded as the source for deep-sea corals and sponges since they do not form symbiosis with Symbiodiniaceae. Non-informative priors were applied, along with both process and residual error terms. To account for minimal trophic discrimination between basal resources and consumer δ^13^C_EAA_ values, we applied a trophic discrimination factor of 0.1 ± 0.1‰ (mean ± SD) as suggested by previous studies^29,36^. Model convergence was assessed using Gelman-Rubin and Geweke diagnostics. Percentage contributions of basal resource groups to consumers were reported in posterior median.

### Reporting summary

Further information on research design is available in the Nature Portfolio Reporting Summary linked to this article.

## Supplementary information

Supporting Information

Description of Additional Supplementary Files

Supplementary Data 1

Reporting Summary

## Data Availability

All the datasets analysed in this study are available in Supplementary Data 1.

## Code availability

All code used in the analyses is available at https://github.com/Qifang-Wj/Wang-et-al.-2025-Communications-Biology.
