# Supplementary material for: Limited contributions of bacteria and fungi to coral nutrition revealed by amino acid δ13C analysis
Source: Commun Biol. 2025 Oct 27;8:1500. doi: 10.1038/s42003-025-08888-x (PMC12559213; doi:10.1038/s42003-025-08888-x)
Supplement: Supplementary file 1 — Supporting Information [file 42003_2025_8888_MOESM1_ESM.pdf]

SUPPORTING INFORMATION

(10 pages, 2 figures and 6 tables)

Limited contributions of bacteria and fungi to coral nutrition revealed by amino acid $\delta^{13}C$ analysis

Qifang Wang[1,2,3,4], Jiachen Li[5], Xijie Zhou[1,3,4], Lingfeng Huang[2], Tuo Shi[6], Tiantian Tang[5,*], Jonathan Y.S. Leung[7,*], Xinqing Zheng[1,3,4,8,*]

[1] *Key Laboratory of Marine Ecology Conservation and Restoration, Third Institute of Oceanography, Ministry of Natural Resources, Xiamen, China*

[2] *Key Laboratory of the Ministry of Education for Coastal and Wetland Ecosystems, College of the Environment and Ecology, Xiamen University, Xiamen, China*

[3] *Observation and Research Station of Island and Coastal Ecosystem in the Western Taiwan Strait, MNR, Zhangzhou, China*

[4] *Observation and Research Station of Wetland Ecosystems in the Beibu Gulf, Ministry of Natural Resources, Xiamen, China*

[5] *State Key Laboratory of Marine Environmental Science, Xiamen University, Xiamen, China*

[6] *Marine Genomics and Biotechnology Program, Institute of Marine Science and Technology, Shandong University, Qingdao, China*

[7] *Guangdong Provincial Key Laboratory of Marine Disaster Prediction and Prevention, Shantou University, Shantou, China*

[8] *Ningbo Institute of Oceanography, Ningbo, China*

[*]Corresponding authors

Email: zhengxinqing@tio.org.cn (Xinqing Zheng); jonathanleung@stu.edu.cn (Jonathan Y.S. Leung); tiantian.tang@xmu.edu.cn (Tiantian Tang)

Abbreviations: Ala, alanine; Gly, glycine; Thr, threonine; Ser, serine; Val, valine; Leu, leucine; Ile, isoleucine; Pro, proline; Asx, asparagine/aspartic acid; Glx, glutamine/glutamic acid; Phe, phenylalanine; Tyr, tyrosine; Lys, lysine; Nor, norleucine; GC-C-IRMS, gas chromatograph coupled to isotope ratio mass spectrometry.

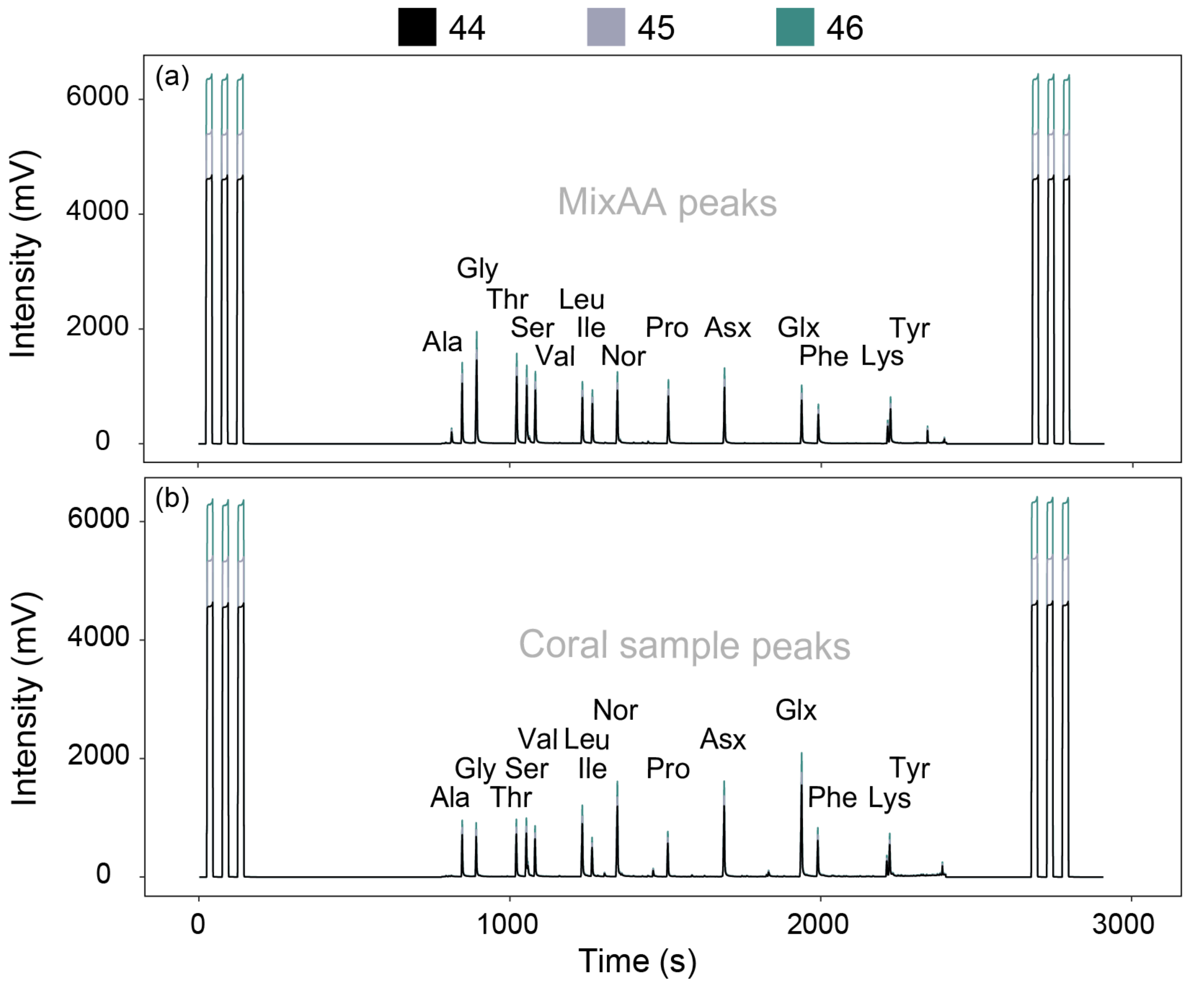

Supplementary Figure 1. GC-C-IRMS chromatograms and mass spectra of **(a)** mixed amino acid (external) standards and **(b)** a coral sample. The numbers in the legend represent atomic masses.

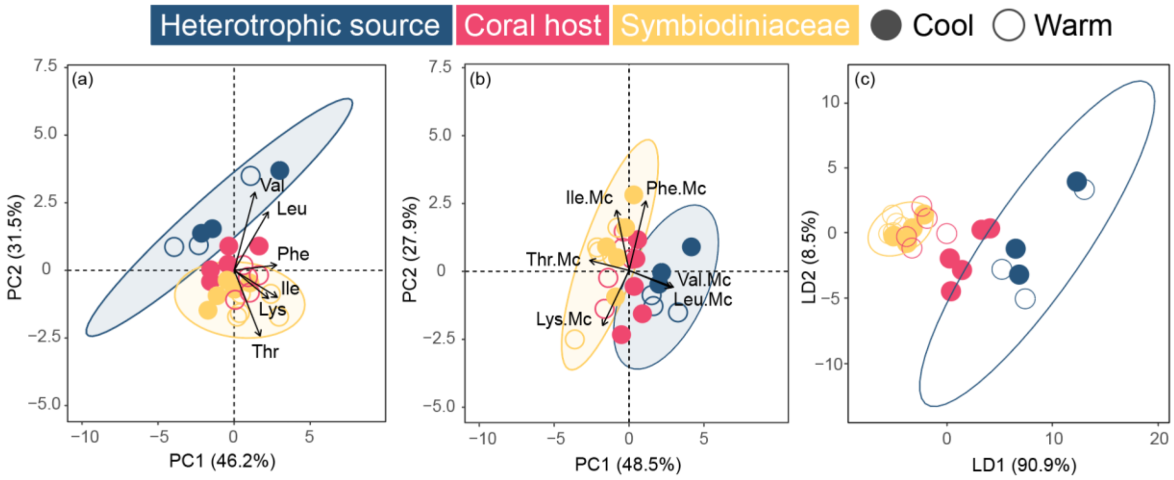

Supplementary Figure 2. Effect of data treatment on multivariate analysis of regional samples. (a) PCA of measured-$\delta^{13}C_{EAA}$ data; (b) PCA of mean-centred $\delta^{13}C_{EAA}$ data; (c) LDA of measured-$\delta^{13}C_{EAA}$ data; LDA of mean-centred $\delta^{13}C_{EAA}$ data; LDA on mean-centred data provides the most distinct separation among basal resource groups, while mean-centring reduces temporal offsets, causing samples from different seasons to cluster more closely.

Supplementary Table 1. The mean (technical replications) stable carbon isotope values ($\delta^{13}C$; ‰) of the amino acids in the coral host and Symbiodiniaceae (shown in paired ID) of *Pocillopora damicornis* colonies as well as the exogenous food sources collected from the Houhai fringe reef: particulate organic matter (POM), sedimentary organic matter (SOM) and zooplankton.

**Fraction**	**Season**	**Ala**	**Thr**	**Val**	**Leu**	**Ile**	**Pro**	**Asx**	**Glx**	**Phe**	**Lys**
Coral 1	Cool	−16.68	−8.46	−26.06	−26.42	−14.43	−10.81	−15.85	−14.89	−22.28	−15.12
Coral_2	Cool	−13.80	−4.38	−21.68	−24.37	−13.38	−10.09	−14.18	−12.78	−21.45	−11.39
Coral_3	Cool	−17.53	−7.88	−25.96	−27.77	−15.84	−10.83	−19.47	−15.15	−24.09	−16.18
Coral_4	Cool	−17.27	−6.61	−27.23	−26.73	−17.65	−11.61	−16.25	−11.79	−25.97	−11.08
Coral_5	Cool	−17.10	−4.19	−23.36	−25.25	−18.43	−10.33	−18.16	−10.45	−22.51	−13.99
Coral_6	Warm	−14.59	−4.57	−24.56	−26.57	−14.82	−10.52	−15.45	−12.66	−20.15	−11.67
Coral_7	Warm	−13.04	−2.02	−24.33	−25.62	−13.19	−5.97	−13.49	−12.00	−19.11	−13.95
Coral_8	Warm	−16.53	−3.39	−26.08	−26.53	−14.27	−11.37	−16.42	−13.60	−20.69	−9.40
Coral_9	Warm	−14.53	−2.07	−24.72	−26.02	−14.16	−8.89	−13.84	−11.81	−20.45	−14.20
Coral_10	Warm	−18.52	−0.94	−26.51	−26.62	−16.05	−10.10	−17.42	−11.17	−23.10	−10.71
Symbiodiniaceae_1	Cool	−18.74	−5.54	−28.02	−28.93	−17.15	−12.36	−13.62	−15.26	−20.70	−16.24
Symbiodiniaceae_2	Cool	−15.28	−0.96	−24.34	−26.71	−14.39	−9.01	−12.00	−13.19	−20.38	−13.09
Symbiodiniaceae_3	Cool	−17.71	−6.14	−27.79	−26.79	−13.97	−10.17	−13.68	−15.40	−21.06	−18.31
Symbiodiniaceae_4	Cool	−17.40	−0.71	−26.06	−26.43	−17.15	−12.84	−12.23	−12.35	−21.80	−12.44
Symbiodiniaceae_5	Cool	−18.10	1.67	−27.94	−28.33	−17.99	−12.43	−11.54	−11.15	−23.72	−19.04
Symbiodiniaceae_6	Warm	−17.14	−1.69	−26.66	−26.73	−14.23	−10.86	−11.57	−14.08	−20.50	−15.48
Symbiodiniaceae_7	Warm	−15.29	−0.13	−25.57	−25.00	−11.68	−9.70	−8.93	−10.83	−19.41	−10.91
Symbiodiniaceae_8	Warm	−16.99	−0.07	−26.74	−28.16	−14.15	−12.26	−10.01	−12.63	−21.60	−13.12
Symbiodiniaceae_9	Warm	−14.66	2.08	−24.88	−25.69	−12.50	−9.12	−8.68	−11.33	−21.10	−2.38
Symbiodiniaceae_10	Warm	−14.07	0.91	−25.50	−28.58	−13.85	−11.86	−11.73	−9.40	−21.70	−13.76
SOM_1	Warm	−14.48	−6.91	−16.02	−20.36	−14.38	−13.13	−13.84	−14.20	−19.34	−11.81
POM_1	Warm	−21.34	−12.58	−24.95	−29.12	−20.21	−20.85	−20.63	−22.36	−27.08	−17.32
Zooplankton_1	Warm	−15.98	−7.52	−24.78	−26.35	−18.68	−16.87	−15.44	−17.68	−25.65	−18.25
SOM_2	Cool	−18.22	−10.19	−16.87	−24.01	−16.24	−15.91	−13.33	−15.45	−18.17	−17.48
POM_2	Cool	−16.54	−10.38	−23.06	−26.87	−18.29	−18.97	−15.71	−16.97	−22.95	−17.05
Zooplankton_2	Cool	−16.90	−10.63	−24.13	−26.58	−18.04	−16.65	−16.30	−14.30	−25.20	−17.56

Supplementary Table 2. Statistical comparison showing the effects of seasonality (i.e., warm vs. cool) and sample fractions (i.e., Symbiodiniaceae (S), exogenous food source (E) and host (H)) on the stable carbon isotope values of the amino acids ($\delta^{13}C_{AA}$). Bold text indicates a significant difference ($p < 0.05$).

Amino acid	Source of variation	*df*	*SS*	*F*	*p*	Pairwise comparison
Ala	Season	1	2.67	0.77	0.39	
	Fraction	2	7.00	1.00	0.38	
	Fraction × Season	2	3.00	0.47	0.63	
	Residual	20	69.38			
Thr	Season	1	24.56	4.32	**0.05**	Warm > Cool
	Fraction	2	159.50	14.04	**< 0.01**	S, H > E
	Fraction × Season	2	2.84	0.25	0.78	
	Residual	20	113.64			
Val	Season	1	0.40	0.06	0.81	
	Fraction	2	31.50	2.62	0.08	
	Fraction × Season	2	3.1	0.26	0.78	
	Residual	20	120.40			
Leu	Season	1	0.10	0.02	0.89	
	Fraction	2	4.50	0.67	0.52	
	Fraction × Season	2	0.90	0.13	0.88	
	Residual	20	67.30			
Ile	Season	1	5.25	1.71	0.21	
	Fraction	2	38.21	6.21	**< 0.01**	S, H > E
	Fraction × Season	2	9.00	1.46	0.26	
	Residual	20	21.25			
Pro	Season	1	4.65	1.28	0.27	
	Fraction	2	114.22	15.76	**< 0.01**	S, H > E
	Fraction × Season	2	1.39	0.19	0.82	
	Residual	20	72.47			
Asx	Season	1	5.31	1.51	0.23	
	Fraction	2	100.88	14.34	**< 0.01**	S > H, E
	Fraction × Season	2	15.05	2.14	0.14	
	Residual	20	70.36			
Glx	Season	1	1.46	0.35	0.56	
	Fraction	2	87.58	10.54	**< 0.01**	S, H > E
	Fraction × Season	2	18.02	2.17	0.14	
	Residual	20	83.09			
Phe	Season	1	16.36	3.56	0.07	
	Fraction	2	24.31	2.64	0.09	
	Fraction × Season	2	18.84	2.05	0.15	
	Residual	20	91.81			
Lys	Season	1	6.13	0.60	0.44	
	Fraction	2	43.23	2.12	0.14	
	Fraction × Season	2	15.04	0.74	0.49	
	Residual	20	203.90			

Supplementary Table 3. Permutational analysis of variance showing the effects of (1) exogenous food source types (particulate organic matter, sedimentary organic matter and zooplankton) on the stable carbon isotope values ($\delta^{13}C$) of 10 amino acids, and (2) sample fractions (combined sources, coral hosts and Symbiodiniaceae), seasons (cool vs. warm) and their interactions on the stable carbon isotope values ($\delta^{13}C$) of 10 amino acids in pooled samples. Bold number indicates significant difference ($p < 0.05$).

**Group**	***df***	***SS***	***R²***	***F***	***p***
(1) Within heterotrophic sources					
Source type	2	18.56	0.58	2.14	0.09
Residual	3	12.96	0.41		
Total	5	31.52	1		
(2) Pooled samples					
Season	1	8.78	0.06	1.97	**0.04**
Fraction	2	40.32	0.27	4.53	**< 0.01**
Season × Fraction	2	8.83	0.06	0.99	0.43
Residual	20	88.95	0.61		
Total	25	146.87	1		

Supplementary Table 4. Eigenvalue and variance (%) of the principal component analysis (Fig. 2).

	Eigenvalue	Variance (%)	Cumulative variance (%)
Dim.1	5.86	53.28	53.28
Dim.2	2.43	22.13	75.41
Dim.3	1.10	10.04	85.45
Dim.4	0.66	6.05	91.49
Dim.5	0.37	3.41	94.91
Dim.6	0.22	2.01	96.92
Dim.7	0.13	1.24	98.15
Dim.8	0.08	0.74	98.89
Dim.9	0.05	0.48	99.38
Dim.10	0.04	0.37	99.75
Dim.11	0.03	0.25	100

Supplementary Table 5. Coefficients of linear discriminants (basal organism groups: Symbiodiniaceae, particulate food sources, fungi and bacteria, Fig. 3). The overall reclassification rate for each source within the defined basal organism groups is 95.42% (organism-specific reclassification rates: 100% for fungi, 95.65% for bacteria, 94.59% for particulate food sources and 96.15% for Symbiodiniaceae).

**Amino acid**	**LD1**	**LD2**	**LD3**
Ile	−0.55	0.19	−0.11
Leu	0.38	−0.15	0.26
Lys	−0.30	−0.49	0.01
Phe	−0.31	0.19	0.43
Thr	−0.20	0.11	0.05
Val	0.11	0.04	0.04

Supplementary Table 6. Coefficients of linear discriminants (Fig. 4). The overall reclassification rate for autotrophic and heterotrophic sources is 100%, including zooplankton, particulate organic matter and sedimentary organic matter.

**Amino acid**	**LD1**	**LD2**	**LD3**
Ile	−0.42	−0.08	0.99
Leu	0.65	1.03	−0.44
Lys	0.08	−0.07	0.16
Phe	−0.54	−0.36	−0.42
Thr	−0.23	0.46	−0.35
Val	1.27	−0.11	0.17
