# Supplementary material for: Limited contributions of bacteria and fungi to coral nutrition revealed by amino acid δ13C analysis
Source: Commun Biol. 2025 Oct 27;8:1500. doi: 10.1038/s42003-025-08888-x (PMC12559213; doi:10.1038/s42003-025-08888-x)
Supplement: Supplementary file 2 — Description of Additional Supplementary Files [file 42003_2025_8888_MOESM2_ESM.pdf]

Description of Additional Supplementary Files

File name: Supplementary Data 1

Description: All the datasets analysed in this study
