# Supplementary material for: Limited contributions of bacteria and fungi to coral nutrition revealed by amino acid δ13C analysis
Source: Commun Biol. 2025 Oct 27;8:1500. doi: 10.1038/s42003-025-08888-x (PMC12559213; doi:10.1038/s42003-025-08888-x)
Supplement: Supplementary file 4 — Reporting Summary [file 42003_2025_8888_MOESM4_ESM.pdf]

Corresponding author(s): Xinqing Zheng, Tiantian Tang, Jonathan Y.S. Leung

Last updated by author(s): Aug 22, 2025

Reporting Summary

Nature Portfolio wishes to improve the reproducibility of the work that we publish. This form provides structure for consistency and transparency in reporting. For further information on Nature Portfolio policies, see our Editorial Policies and the Editorial Policy Checklist.

Statistics

For all statistical analyses, confirm that the following items are present in the figure legend, table legend, main text, or Methods section.

n/a	Confirmed	
☐	☒	The exact sample size (*n*) for each experimental group/condition, given as a discrete number and unit of measurement
☐	☒	A statement on whether measurements were taken from distinct samples or whether the same sample was measured repeatedly
☐	☒	The statistical test(s) used AND whether they are one- or two-sided<br>*Only common tests should be described solely by name; describe more complex techniques in the Methods section.*
☒	☐	A description of all covariates tested
☐	☒	A description of any assumptions or corrections, such as tests of normality and adjustment for multiple comparisons
☐	☒	A full description of the statistical parameters including central tendency (e.g. means) or other basic estimates (e.g. regression coefficient) AND variation (e.g. standard deviation) or associated estimates of uncertainty (e.g. confidence intervals)
☐	☒	For null hypothesis testing, the test statistic (e.g. *F*, *t*, *r*) with confidence intervals, effect sizes, degrees of freedom and *P* value noted<br>*Give P values as exact values whenever suitable.*
☒	☐	For Bayesian analysis, information on the choice of priors and Markov chain Monte Carlo settings
☒	☐	For hierarchical and complex designs, identification of the appropriate level for tests and full reporting of outcomes
☒	☐	Estimates of effect sizes (e.g. Cohen's *d*, Pearson's *r*), indicating how they were calculated

Our web collection on statistics for biologists contains articles on many of the points above.

Software and code

Policy information about availability of computer code

Data collection	No software was used for data collection.
Data analysis	The figures were created and analysed using an open-source software R version 4.0.5.

For manuscripts utilizing custom algorithms or software that are central to the research but not yet described in published literature, software must be made available to editors and reviewers. We strongly encourage code deposition in a community repository (e.g. GitHub). See the Nature Portfolio guidelines for submitting code & software for further information.

Data

Policy information about availability of data

All manuscripts must include a data availability statement. This statement should provide the following information, where applicable:

- Accession codes, unique identifiers, or web links for publicly available datasets
- A description of any restrictions on data availability
- For clinical datasets or third party data, please ensure that the statement adheres to our policy

The data analysed in the present study are available in the supplementary information.

Research involving human participants, their data, or biological material

Policy information about studies with human participants or human data. See also policy information about sex, gender (identity/presentation), and sexual orientation and race, ethnicity and racism.

Reporting on sex and gender	This work does not involve human research participants.
Reporting on race, ethnicity, or other socially relevant groupings	N/A
Population characteristics	N/A
Recruitment	N/A
Ethics oversight	N/A

Note that full information on the approval of the study protocol must also be provided in the manuscript.

Field-specific reporting

Please select the one below that is the best fit for your research. If you are not sure, read the appropriate sections before making your selection.

☐ Life sciences ☐ Behavioural & social sciences ☒ Ecological, evolutionary & environmental sciences

For a reference copy of the document with all sections, see nature.com/documents/nr-reporting-summary-flat.pdf

Ecological, evolutionary & environmental sciences study design

All studies must disclose on these points even when the disclosure is negative.

Study description	We investigated the nutrient sources of reef-building corals (algal symbionts and heterotrophic food sources) across different seasons using amino acid carbon isotope analysis. This study employed a factorial experimental design with two factors: season and sample fraction (coral host, symbionts, and heterotrophic food sources). In addition to experimental data, published datasets were incorporated into the analysis. A linear discriminant analysis was performed to quantify bacterial and fungal contributions to coral nutrition, providing a more comprehensive understanding of their nutrient acquisition strategies.
Research sample	We selected Pocillopora damicornis as the model organism for this study due to its ecological significance, wide distribution across the Pacific and Indian Oceans, and flexibility in nutritional strategies. This mixotrophic coral is ideal for investigating autotrophic and heterotrophic nutrient acquisition. The samples were collected from healthy colonies in the northern South China Sea to represent natural populations within this region. Additionally, we incorporated existing datasets, including amino acid carbon isotope values ($\delta^{13}C_{EAA}$) for primary producers (e.g., zooxanthellae, POM, fungi and bacteria) and various animal consumers, to supplement our analysis. These datasets were sourced from previously published studies and used to estimate bacterial and fungal contributions to coral nutrition. Detailed information about sample origins, taxa, and data sources is provided in the main text and supplementary materials.
Sampling strategy	Each sample type per season included at least five biological replicates to ensure statistical robustness.
Data collection	Experimental data were collected by the first and second authors following established protocols, while existing datasets were collected from published studies cited in the manuscript.
Timing and spatial scale	All the samples were collected from the Houhai fringe reef at a depth of 2 m in November 2019 and June 2020, and stable isotope analysis was conducted in 2022.
Data exclusions	No data was excluded from the analyses.
Reproducibility	All attempts to repeat this experiment were successful.
Randomization	The colonies were healthy and spatially separated from each other by at least 5 m and randomly collected.
Blinding	All experiments followed established protocols.

Did the study involve field work? ☒ Yes ☐ No

Field work, collection and transport

Field conditions	From November 2019 to June 2020, there was a general rise in seawater temperature from 22 °C to 28 °C, while chlorophyll concentration (Chl a) decreased from approximately 4 to 2 mg m-3.
Location	Houhai fringe reef (18.276°N, 109.726°E) in the southern coast of Hainan Island, China. Currently, this reef is designated as a refuge for corals due to the cooler upwelling in summer. Over 50 coral species (~50% benthic cover) have been documented in this reef, with Pocillopora damicornis as a dominant species.
Access & import/export	All the samples were collected by SCUBA divers, flash-frozen in liquid nitrogen and stored at −20 °C until analysis. All collections were under the permission of the local government and Third Institute of Oceanography.
Disturbance	No disturbance was caused by the study.

Reporting for specific materials, systems and methods

We require information from authors about some types of materials, experimental systems and methods used in many studies. Here, indicate whether each material, system or method listed is relevant to your study. If you are not sure if a list item applies to your research, read the appropriate section before selecting a response.

Materials & experimental systems

n/a	Involved in the study
☒	☐ Antibodies
☒	☐ Eukaryotic cell lines
☒	☐ Palaeontology and archaeology
☐	☒ Animals and other organisms
☒	☐ Clinical data
☒	☐ Dual use research of concern
☒	☐ Plants

Methods

n/a	Involved in the study
☒	☐ ChIP-seq
☒	☐ Flow cytometry
☒	☐ MRI-based neuroimaging

Animals and other research organisms

Policy information about studies involving animals; ARRIVE guidelines recommended for reporting animal research, and Sex and Gender in Research

Laboratory animals	The study did not involve laboratory animals.
Wild animals	P. damicornis were collected across seasons by SCUBA divers using a hammer and chisel and frozen in liquid nitrogen directly.
Reporting on sex	No
Field-collected samples	This study did not maintain field-collected animals in the laboratory.
Ethics oversight	The Third Institute of Oceanography, Ministry of Nature Resources, China

Note that full information on the approval of the study protocol must also be provided in the manuscript.

Plants

Seed stocks	N/A
Novel plant genotypes	N/A
Authentication	N/A
